# Regulatory links between imprinted genes: evolutionary predictions and consequences

**DOI:** 10.1098/rspb.2015.2760

**Published:** 2016-02-10

**Authors:** Manus M. Patten, Michael Cowley, Rebecca J. Oakey, Robert Feil

**Affiliations:** 1 Department of Biology, Georgetown University, 37th and O Streets NW, Washington, DC 20057, USA; 2 Center for Human Health and the Environment, Department of Biological Sciences, North Carolina State University, Raleigh, NC 27695, USA; 3 Department of Medical and Molecular Genetics, King's College London, London SE1 9RT, UK; 4 Centre National de Recherche Scientifique (CNRS), Institute of Molecular Genetics (IGMM), 1919 route de Mende, Montpellier 34293, France; 5 University of Montpellier, 163 rue Auguste Broussonnet, Montpellier 34090, France; 6 Medical Institute of Bioregulation, Kyushu University, 3-1-1 Maidashi, Higashi-ku, Fukuoka 812-8582, Japan

**Keywords:** evolution, genomic imprinting, gene network, *trans* regulation

## Abstract

Genomic imprinting is essential for development and growth and plays diverse roles in physiology and behaviour. Imprinted genes have traditionally been studied in isolation or in clusters with respect to *cis*-acting modes of gene regulation, both from a mechanistic and evolutionary point of view. Recent studies in mammals, however, reveal that imprinted genes are often co-regulated and are part of a gene network involved in the control of cellular proliferation and differentiation. Moreover, a subset of imprinted genes acts *in trans* on the expression of other imprinted genes. Numerous studies have modulated levels of imprinted gene expression to explore phenotypic and gene regulatory consequences. Increasingly, the applied genome-wide approaches highlight how perturbation of one imprinted gene may affect other maternally or paternally expressed genes. Here, we discuss these novel findings and consider evolutionary theories that offer a rationale for such intricate interactions among imprinted genes. An evolutionary view of these *trans*-regulatory effects provides a novel interpretation of the logic of gene networks within species and has implications for the origin of reproductive isolation between species.

## 1. Genomic imprinting in development

Genomic imprinting is a mechanism of gene regulation whereby genes are transcribed from either the maternally or the paternally inherited allele. In contrast to the majority of genes that are expressed from both the parental chromosomes, this is the property of an exclusive minority of a few hundred genes both in seed plants and mammals [1–3]. Imprinting evolved convergently in the two groups and much progress has been made on understanding the underlying mechanisms [4]. In the current review, we concern ourselves primarily with the data from mammals, though theoretical aspects of our discussion can be generalized to imprinting in plants—and perhaps also social insects [5].

Imprinted genes are frequently, but not always, organized into clusters of coordinately regulated genes [6]. Although hundreds of protein-coding genes and non-coding RNAs (ncRNAs) are controlled by genomic imprinting in eutherians, only eight of these, all involved in growth, are imprinted also in metatherians (marsupials), with no evidence for parental-origin-specific, mono-allelic gene expression in monotremes (e.g. platypus) [7]. Thus, genomic imprinting probably evolved in a therian ancestor, when the extra-embryonic lineage became essential for offspring development and with the emergence of extensive maternal contributions to postnatal development.

Genomic imprinting provides a dosage regulatory mechanism that has been demonstrated to be important for normal embryonic growth and development via detailed genetic, epigenetic and phenotypic dissection of mouse mutant models [8,9] and through studies on congenital imprinting disorders in humans (e.g. [10]). A number of postnatal phenotypes are attributed also to improper imprinted gene regulation, including diabetes, obesity, mental retardation, feeding behaviour and cancer. The biochemical systems underpinning these phenotypes control cell signalling, nutrient transport, metabolism, protein synthesis and the action of transcription factors [11].

The epigenetic mechanisms governing the parental-origin-specific, mono-allelic expression of imprinted genes involve differential DNA methylation marks [12]. Using knockout mouse models, ‘imprinting control regions’ (ICRs) have been identified that confer on the two parental alleles of an imprinted locus different DNA methylation states, which are inherited from the germ line to provide a stable parental-origin-specific mark on each allele in the embryo. There is growing evidence to also support contributions from post-translational histone modifications in imprinted gene regulation [13].

The mechanisms that give rise to imprinted gene expression have been studied in depth at numerous imprinted loci, particularly in mice, and imprinting provides an ideal paradigm to compare the active and silent alleles at a single locus [14]. Well-known mechanisms include the action of small and long non-coding RNAs (lncRNAs) working *in cis* to regulate gene expression at imprinted loci [15,16], while others employ allele-specific differential binding of the methylation-sensitive insulator protein CTCF, known as the ‘enhancer competition mode’ of gene regulation [17,18].

Here, we review other levels of imprinted gene regulation that are less well understood—namely, the co-regulation of imprinted genes in an ‘imprinted gene network’ (IGN) [19] and how several imprinted genes modify the expression of others *in trans*. We interpret these phenomena in the light of the main theories of the evolution of genomic imprinting, which we first review below.

## 2. Evolutionary theories for imprinted gene expression

Among various theories to explain the evolution of genomic imprinting (reviewed in [20,21]) two have attracted broad attention: the ‘kinship theory’, proposed by Haig [22], and the ‘maternal–offspring co-adaptation theory’, proposed by Wolf and co-workers [23–25].

The kinship theory of imprinting—often referred to as the parental conflict theory—views the modulation of gene expression levels as the ultimate function of imprinting. It holds that imprinting evolved because of the opposing effects that modifying a gene's dosage can have on the fitness of one's mother's and father's kin. Within an individual, the maternally- and paternally derived alleles of a gene can, therefore, ‘disagree’ over the optimal expression level: increasing the total dosage might increase one's maternal kin's fitness and reduce one's paternal kin's fitness, or vice versa. Under the kinship theory, complete imprinted silencing is the expected resolution to this within-gene conflict, and the maternally expressed genes (called ‘MEGs’) and the paternally expressed genes (called ‘PEGs’) that result are expected to express at the maternally or paternally derived allele's optimal level, respectively [26].

The co-adaptation theory [23] sees imprinting as a way to choose which of the two alleles at a locus to express. It rests on the idea that one of the two alleles confers higher fitness because of its epistatic interactions with other genes, which may reside in other individuals or within the same individual [25]. Here ‘epistasis’ is used in the statistical sense, which is common usage in population genetics, in contrast to the biochemical sense, which envisions genes residing in a shared molecular pathway [27]. When natural selection acts on such epistatic interactions between genes, populations become enriched for haplotypes that associate favourably interacting—or ‘co-adapted’—alleles [28]. Imprinting is predicted to evolve under two different scenarios. First, a mother's genotype is more likely to have a relatively high-fitness interaction with her offspring's maternally inherited than with its paternally inherited haplotype, as the offspring's maternally inherited haplotype is enriched for alleles that interact well with the alleles of the mother's genotype. This favours the imprinted silencing in the offspring of the paternally inherited alleles of genes involved in these interactions [23]. Second, one expects that the allelic interactions within any particular haplotype—e.g. allelic interactions within the paternally derived haplotype—will produce higher fitness for their bearers, on average, than will the interactions of alleles chosen from opposite parental origins. This will be especially true for genes that are physically linked on a chromosome, because tight linkage prevents recombination from breaking up favourably interacting alleles. This can select for imprinted expression of a gene that interacts epistatically with an imprinted gene [24].

## 3. Network(s) of coordinately expressed imprinted genes

Earlier developmental studies used chromosomal-translocation mouse lines to generate maternal and paternal uniparental disomies, or duplications, for individual chromosomes or chromosomal regions. Besides unravelling specific roles of imprinting, these studies provided evidence for phenotypic cross-talk between different imprinted chromosomal domains [29]. Subsequent research showed that many imprinted genes are functionally related and part of common pathways. The most striking example of this is provided by the insulin (INS)–insulin-like growth factor (IGF) signalling pathway, which comprises the imprinted IGF2, IGF2R, INS2 and the growth factor receptor binding protein GRB10 encoding genes. This pathway controls cellular proliferation and growth. Other biological functions that involve multiple imprinted genes include nutrient and ion transport, extracellular matrix control, and protein synthesis and degradation [11].

Insights into common roles and co-regulation have also emerged from studies on human imprinting disorders. Although linked to genetic or epigenetic changes at individual imprinted loci, some of these complex disorders show considerable clinical overlap, with frequent occurrence of common co-morbidities including aberrant growth, obesity and type-2 diabetes [30].

Genes within individual imprinted domains often show similar developmental patterns of expression. Furthermore, imprinted genes become upregulated in concert at different domains upon differentiation, particularly in brain and placental development [11,31]. Systems biology approaches have confirmed that many imprinted genes are indeed co-regulated in their expression levels. Through comparison of tissue-specific gene expression datasets, initially some 15 co-regulated imprinted genes were pinpointed [19]. Comprehensive follow-up studies indicated that the imprinted gene network comprises at least 60 imprinted genes as well as many non-imprinted genes [32,33]. In agreement with earlier studies [34,35], in fibroblastic cells forced to exit the cell cycle through removal of serum from the culture medium, all imprinted genes analysed became strongly upregulated, and the same was observed for many non-imprinted genes that are part of the network [32]. Concordantly, ectopic overexpression of imprinted genes reduced the proliferation rate of cultured fibroblasts. Conversely, when cells were induced to re-enter the cell cycle, expression of the imprinted genes was strongly downregulated. Similar observations were made in an *in vivo* model of induced muscle regeneration and differentiation [32]. MEGs and PEGs behaved similarly in the cell-based and the *in vivo* tissue regeneration studies.

The new data evoke an intricate network of imprinted genes involved in cell-cycle exit and differentiation. Whether the structure of this network, which also comprises many non-imprinted extracellular matrix genes, is comparable between different cell types, or whether different networks exist, is not yet known. Regardless, the idea of a coordinately regulated network of imprinted genes is gaining traction.

In addition, besides the different *cis*-regulatory actions of the products of imprinted loci—including the often repressive role of imprinted lncRNAs—recent studies show that the products of imprinted loci can directly regulate other imprinted genes *in trans*. Empirical examples are reviewed below in ‘*Cis* and *trans* regulation by imprinted genes: testing the evolutionary predictions’. These observations, combined with the notion of an imprinted gene network, urge us to consider what the main evolutionary theories predict about the interactions between—and the interdependence of—imprinted genes.

## 4. Evolutionary predictions for the interactions between—and interdependence of—imprinted genes

The ‘kinship’ (or ‘parental conflict’) theory [22] and the ‘co-adaptation’ theory [23] are germane to a discussion of the imprinted gene network and the *trans*-regulatory effects of imprinted genes. Both theories were originally formulated to explain the evolutionary origin of imprinted silencing *in cis*, but extensions to *trans*-regulatory interactions, which follow naturally, have since been made [24,25,36,37]. As described above, the kinship theory [22] of genomic imprinting centres on the conflict between alleles of maternal and paternal origin over a gene's expression level. But as the conflict over expression level *within* a locus is resolved by imprinted expression *in cis*, it creates a second conflict *between* loci over the same issue. This is because the level of expression that an MEG or a PEG will evolve to is necessarily higher than is optimal for most other genes in the genome. All paternally derived alleles in a genome would experience higher fitness if MEGs were to reduce their expression, and vice versa for maternally derived alleles and PEGs [36,38].

**Figure 1.**
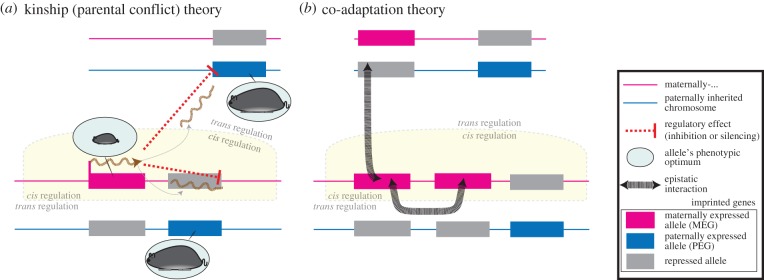
Predicted *cis* and *trans* effects in light of the kinship and co-adaptation theories. (*a*) Under the kinship theory, MEGs and PEGs have different phenotypic optima (shown in light blue ovals). Shown here for ease of presentation are the growth optima, but other phenotypes—most notably phenotypes associated with social behaviour—are subject to similar conflicts between the two parental genomes. As predicted by the theory [22], MEGs typically inhibit growth and PEGs enhance it. Shown here is an MEG that achieves growth inhibition via an ncRNA both *in cis* (within the defined region) and *in trans* (outside of the defined locus). In *cis*, the ncRNA is involved in silencing the neighbouring gene, a PEG, thereby reducing the total level of expression of a growth enhancing gene. In *trans*, the ncRNA reduces the total expression level of a different PEG, further reducing the total gene expression level of growth enhancing genes. Similar effects on gene expression are predicted for MEGs that encode proteins. (*b*) Under the co-adaptation theory [23–25], imprinting is favoured by natural selection to coordinate the expression of genes that interact epistatically. Epistatic interactions between alleles within a parental haplotype are fitter, on average, than epistatic interactions that would be produced between parental haplotypes. This results from selection in the prior generation, which produces an excess of haplotypes with good epistatic combinations. Imprinting can, therefore, spread from one imprinted gene to its epistatic partners so as to increase the chance of a high-fitness interaction. This spread is more likely for linked genes, leading to a prediction of imprinted genes with *cis*-regulatory effects on other imprinted genes. Additionally, unlinked genes are subject to the same selective pressure, giving rise to the predicted parental-origin-specific pattern of *trans-*regulatory effects.

Here we apply this evolutionary logic to predict the nature of the *trans*-regulatory interactions between imprinted genes. Under the kinship theory, the expectation of *trans*-regulatory effects stems from the between-gene conflict mentioned above. A hypothetical example illustrates the basic premise. Suppose that a gene unlinked to the paternally expressed *Igf2* is capable of modifying the expression level of *Igf2 in trans*. If increased expression of the *trans*-regulator gene results in decreased expression of *Igf2*, then the maternally derived allele of the *trans* regulator will favour greater expression than the paternally derived allele will. This disagreement between maternally and paternally derived alleles at the *trans*-regulator gene is yet another within-gene conflict over total expression level, and, consequently, the prediction is for imprinted silencing of the paternally derived allele [26]. We therefore expect that MEGs with direct *trans*-regulatory effects will reduce the gene expression level of PEGs, and *vice versa* for PEGs with *trans*-regulatory effects on MEGs (table 1 and figure 1). The underlying logic for our prediction is not new—we are simply extending the kinship theory to a novel source of within-gene conflict. In fact, it is analogous to the way the kinship theory explains the reciprocal imprinting pattern of genes such as *Igf2* and *Igf2r*, whose products have opposing effects on the phenotype [39].

**Table 1.** Predictions from two evolutionary theories (‘kinship’ and ‘co-adaptation’) for the outcome of knockout or overexpression studies with imprinted genes.

experimental treatment	predictions from kinship theory	predictions from co-adaptation theory
MEG knockout/knockdown	MEG expression level down *in cis* PEG expression level up *in cis* and *trans*	expression levels of MEGs more perturbed than expression levels of PEGs *in cis* and *trans* enrichment of MEGs among suite of genes showing effects
PEG knockout/knockdown	PEG expression level down in *cis* MEG expression level up *in cis* and *trans*	expression levels of PEGs more perturbed than expression levels of MEGs *in cis* and *trans* enrichment of PEGs among suite of genes showing effects
MEG overexpression	PEG expression level down *in cis* and *trans*	expression levels of MEGs more perturbed than expression levels of PEGs *in cis* and *trans* enrichment of MEGs among suite of genes showing effects
PEG overexpression	MEG expression level down *in cis* and *trans*	expression levels of PEGs more perturbed than expression levels of MEGs *in cis* and *trans* enrichment of PEGs among suite of genes showing effects

**Figure 2.**
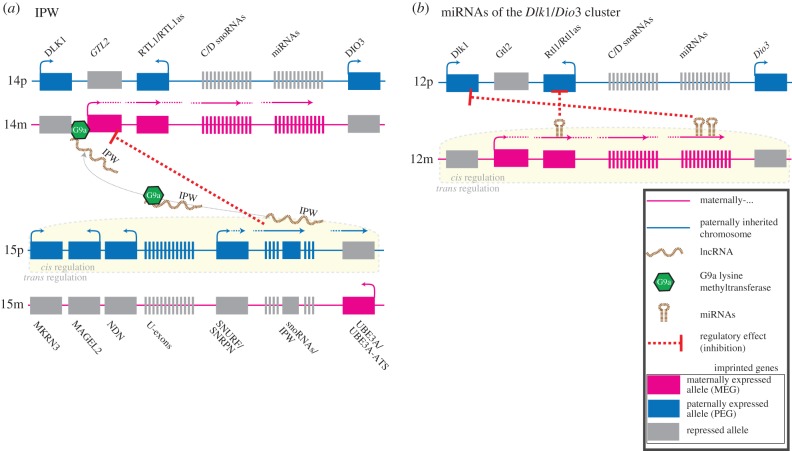
(*a*) Effects of *IPW* lncRNA *in trans*. *IPW*, a PEG in the PWS imprinted domain of human chromosome 15, produces an lncRNA that has inhibitory effects on the expression of several MEGs in the imprinted *DLK1*-*DIO3* domain on chromosome 14. *IPW* appears to achieve its *trans* regulation by recruiting G9A, a lysine methyltransferase that modifies chromatin, to the *DLK1*-*DIO3* region [41]. (*b*) The *Dlk1*-*Dio3* locus on mouse chromosome 12 is host to imprinted genes with *trans* effects. The expression of *Rtl1*, a PEG, is regulated by miRNAs processed from the *Rtl1as*, a maternally expressed antisense RNA. The expression of these miRNAs reduces the expression of *Rtl1* [42]. Similarly, in postnatal muscle in the mouse, a cluster of maternally expressed miRNAs located further downstream appears to negatively affect *Dlk1* expression [43].

One of the seemingly paradoxical findings in the empirical studies on the imprinted gene network [19,32] is that both MEGs and PEGs are coordinately upregulated and downregulated. This appears to run counter to the kinship theory's expectation that MEGs and PEGs will exert opposing effects on each other's expression levels. We suggest one possible explanation for this coordinate regulation, speculative as it may be: conflict of the sort described above [37]. In other words, concomitant expression of MEGs and PEGs is exactly what one predicts when such genes are in conflict. If the products of MEGs and PEGs have antagonistic effects on phenotype (e.g. *Igf2* and *Igf2r*), then any signal that upregulates gene expression for one will come to be used by the other to upregulate itself. Under the assumption that maternally and paternally derived genomes have different optimal phenotypes, any time a signal causes a PEG to be upregulated, an MEG with antagonistic phenotypic effects should evolve to ‘eavesdrop’ for that signal and respond with upregulation itself. Consequently, as an organism produces or receives such a signal, both MEGs and PEGs will respond in concert. We suggest that regulatory eavesdropping of this sort might help to explain why imprinted genes with opposite patterns of expression cluster. Residing in the same chromosomal domain might help imprinted antagonists, such as the microRNAs (miRNAs) of the *Dlk1*-*Dio3* domain (see below), coordinate their expression with that of their targets, producing the *trans*-homologue effects that are seen at several imprinted gene clusters [40] (figure 2). At an even more specific level of coordination, MEGs and PEGs with opposing effects on a single phenotype can share enhancers, as is the case for *H19* and *Igf2* [44].

Note that correlated expression patterns do not in any way contradict the idea that there are MEGs and PEGs with antagonistic effects on each other's expression levels, and single-gene knockout experiments, of the type reviewed in the next section, are well suited to isolate this type of *trans* effect. One testable prediction that follows from our model of network evolution is that vertebrates with similar developmental programmes but without imprinting (e.g. zebrafish) would not evolve similar patterns of coordinated regulation of the homologous genes. Where there is no imprinting, the presumption is that there are no conflicts of interest between parental genomes, and genes with antagonistic effects on phenotype (like *Igf2* and *Igf2r*) would not be ‘listening in’ on the signals used to regulate other genes.

The co-adaptation theory predicts that when an imprinted gene epistatically interacts with a second, bi-allelically expressed gene within the same individual, the latter will evolve imprinted silencing in a matching direction to the first [24] (figure 1). Because of the associations that selection builds between interacting loci, the interaction between two co-inherited alleles is likely to be more adaptive for its bearer than an interaction involving alleles inherited from different parents. This creates selection pressure to silence one allele at a locus in a parental-origin-specific fashion. The theory is sufficiently general to accommodate various modes of interaction between genes: one interpretation could be that the genes encode proteins that interact during development; another is that genes could interact at the transcriptional level [24]. The predictions that follow from the co-adaptation theory are straightforward (table 1). Physical linkage on the same chromosome increases the likelihood that interacting genes will evolve the same direction of imprinted silencing and expression. With respect to predictions about *trans* modification of gene expression levels, if imprinted genes have such effects, and if such interactions are subject to selection, the co-adaptation theory predicts that the interacting genes would be expressed from the same parental genome. The theory does not, however, predict the direction of gene expression level modification in such *trans* interactions. For example, in studies where an MEG is knocked out or over-expressed, the co-adaptation theory predicts that the gene expression levels of other MEGs, which are in the same network, are more likely to be affected (or dysregulated) than those of PEGs, but it does not offer a prediction of whether these other MEGs will be upregulated or downregulated. With respect to imprinted gene networks and the coordinate regulation of sets of imprinted genes, the co-adaptation theory predicts separate networks for MEGs and PEGs [24].

## 5. *Cis* and *trans* regulation by imprinted genes: testing the evolutionary predictions

Imprinted nuclear lncRNAs regulate gene expression through diverse mechanisms, both *in cis* and *in trans*. One mechanism of *in cis* action of non-coding RNA is exemplified by the imprinted *Airn* lncRNA at the *Igf2r* locus. *Airn* is expressed from the paternal allele of the ICR, and its transcription interferes with recruitment of RNA polymerase-II to the *Igf2r* promoter, thus ensuring the imprinted expression of *Igf2r* from the maternally inherited allele only [15]. In the embryo proper, the sequence of the lncRNA transcript seems not to be critical, as the process of transcription itself is sufficient to silence *Igf2r in cis* [45]*.* In the placenta, however, the *Airn* lncRNA mediates the recruitment of the lysine methyltransferase G9A (also called EHMT2, KMT1C) to other genes of the domain, which then become repressed on the paternal allele [46]. Other imprinted lncRNAs that regulate chromatin *in cis* include *Kcnq1ot1,* at the growth-related *Kcnq1* imprinted domain, the *Nespas* lncRNA at the *Gnas* imprinted locus, and possibly also the *Gtl2* (also called *Meg3*) lncRNA at the *Dlk1*-*Dio3* imprinted domain [16,47,48]. In the mouse placenta, *Kcnq1ot1* lncRNA mediates the recruitment *in cis* of Polycomb repressive complexes and G9A [49,50]. This contributes to the allelic repression of several imprinted genes of this domain. A simple picture emerges: *cis*-acting imprinted lncRNAs (or their transcription) are involved in the imprinted repression of neighbouring genes on the same parental chromosome. In the examples above, paternally expressed lncRNAs are involved in repression on the paternal chromosome, thus giving rise to MEGs. Achieving imprinted expression in this way (i.e. by *cis* silencing) is strongly predicted by the kinship theory. This pattern is also consistent with the co-adaptation theory.

There is a growing appreciation for the idea that besides their well-characterized roles in *cis* regulation, imprinted lncRNAs may also function *in trans* and thus have the potential to regulate many loci across multiple chromosomes. One example is *IPW*, which regulates imprinted gene loci *in trans* by interacting with G9A (figure 2). *IPW* is expressed from the paternally inherited allele of the Prader–Willi syndrome (PWS) locus on human chromosome 15 [51]. Induced pluripotent stem cells (iPSCs) derived from PWS patients, which lack expression of *IPW*, exhibit elevated expression of the maternally expressed non-coding RNA genes at the *DLK1-DIO3* locus on chromosome 14 [41]. The expression of these MEGs can be restored to near wild-type levels by overexpression of *IPW in trans*, identifying the imprinted lncRNA as the regulator of an imprinted gene network. Through recruitment of G9A, *IPW* appears to modulate histone H3 lysine-9 methylation at the ICR of the *DLK1-DIO3* domain. This would explain how the paternally expressed lncRNA *IPW* affects expression of all the maternally expressed ncRNAs (miRNAs, snoRNAs and lncRNAs) of this domain, a pattern of *trans* regulation predicted by the kinship theory, but inconsistent with the co-adaptation theory.

**Figure 3.**
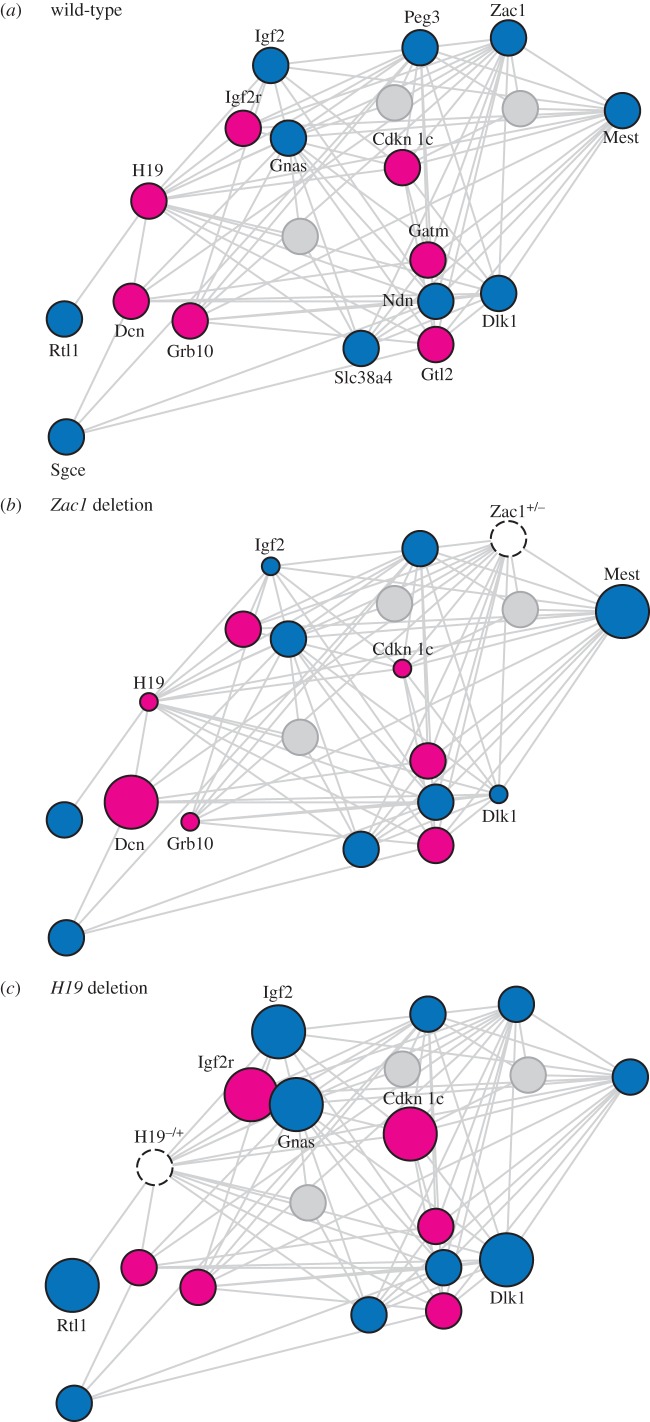
Roles of *Zac1* and *H19* in the imprinted gene network. (*a*) The imprinted gene network, as defined by Gabory *et al.* [52] and Varrault *et al*. [19]. MEGs are shown in pink, PEGs in blue. Grey circles indicate that the network also consists of non-imprinted genes; three are shown for illustrative purposes only. The network also consists of other imprinted genes, but for clarity only those with the strongest interactions are shown. (*b*) The deletion of the paternally inherited allele of *Zac1* in fetal mouse liver affects the expression levels of several genes. Circle sizes indicate relative expression levels. Only genes with significant expression changes are labelled. (*c*) Deletion of the maternally inherited allele of *H19* in fetal mouse muscle also influences the expression of several genes in the IGN.

Using a mouse model of *H19* deletion, Gabory *et al.* [52] showed that loss of the imprinted *H19* lncRNA (an MEG) engendered an upregulation of a network of at least six imprinted genes, both MEGs and PEGs, residing on six different chromosomes (figure 3). Wild-type expression levels were restored following overexpression of *H19 in trans*, indicating that the lncRNA itself is important for this function, rather than its transcription. *H19* transcripts recruit the methyl-CpG-binding domain protein MBD1 to the differentially methylated regions (DMRs) associated with at least some of the imprinted genes in this network [53]. It is through interaction with histone H3 lysine-9 methyltransferase that the *H19* lncRNA-MBD1 complex extensively modulates imprinted gene expression. The similar repressive effects of *H19* lncRNA on the expression of MEGs and PEGs are evolutionarily unexpected. However, the five genes that Monnier *et al.* [53] showed to be directly targeted by both *H19* and MBD1 comprise four PEGs and one MEG that is not imprinted at the stage of development they studied, in line with the predictions of the kinship theory. The co-adaptation theory predicts an effect of *H19* on MEGs only (table 1).

Other imprinted genes use their protein products to perform a *trans*-regulatory function. Murine *Peg3* encodes a protein that binds to DNA through its zinc-finger motifs [54]. ChIP experiments have identified numerous PEG3 binding sites in the genome, including at imprinted genes [54,55]. Of note, PEG3 binds the maternally expressed *Zim1* gene, and mouse models of *Peg3* deletion exhibit elevated *Zim1* transcript levels, suggesting that the normal function of PEG3 is to repress *Zim1* expression [55]. This is predicted by the kinship, but not the co-adaptation, theory.

PEG3 also binds to the mouse *Grb10* locus, an imprinted gene that is expressed from the maternal allele in many tissues, but not in brain, where its expression is from the paternal allele only [56]. In neonatal brain isolated from *Peg3* mutant mice, expression of *Grb10* is reduced relative to wild-type controls [54]. Thus, PEG3 promotes expression of *Grb10* in neonatal brain, where this gene is paternally expressed. This PEG–PEG interaction is consistent with the co-adaptation theory. PEG–PEG interactions are not strongly predicted by the kinship theory, though the direction of the effect on gene expression levels—a PEG that enhances the expression of another PEG—is not inconsistent with the theory. The effect of PEG3 on *Grb10* expression in other embryonic mouse tissues, where *Grb10* is expressed from the maternal chromosome, is unclear.

The imprinted transcription factor PLAGL1 (also called ZAC1) binds to DNA through its zinc-finger domains and regulates other imprinted genes [19] (figure 3). *Plagl1*/*Zac1* is a PEG and studies in the mouse show that it promotes expression of both MEGs and PEGs, including *Igf2*, *H19*, *Kcnq1ot1*, *Cdkn1c* and *Dlk1* [19,57]. The protein binds directly to an enhancer that controls both *Igf2* and *H19* expression, and to the promoter of *Kcnq1ot1*, the lncRNA that controls the imprinted *Kcnq1* domain [19]. Perturbation of PLAGL1's activating role is causally involved in transient neonatal diabetes mellitus (TNDM) [58]. Reduction of *PLAGL1* expression in the human placenta is linked to intra-uterine growth restriction and correlates with reduced expression of *IGF2* and *H19*, and of non-imprinted metabolic genes in the same network [59]. Combined, these effects of PLAGL1 on the imprinted gene network are not directly predicted by either the kinship or the co-adaptation theory (table 1).

Whereas *Plagl1* encodes a transcription factor, the precise mechanism of PEG3's action remains unclear. Like the lncRNAs *H19* and *IPW*, PEG3 seems to interact with lysine methyltransferases and could thus affect chromatin regulation at target genes [55]. Other imprinted regulators of IGNs function through entirely different mechanisms. At the mouse imprinted *Dlk1-Dio3* domain, for example, *Rtl1*/*Peg11* mRNA levels are influenced by miRNAs processed from the maternally expressed *Rtl1as/antiPeg11* transcript [42,60] (figure 2). These imprinted miRNAs guide RISC-mediated cleavage of *Rtl1*/*Peg11* mRNA, thereby functioning to repress *Rtl1*/*Peg11* at the post-transcriptional level. Similarly, maternally expressed miRNAs in this cluster seem to control the expression of the paternally expressed protein-coding gene *Dlk1* in postnatal muscle [43]. Thus, through the action of miRNAs, MEGs reduce the expression of PEGs. This is consistent with the kinship theory's expectation of conflict between the maternally and paternally inherited genomes.

Indirect regulatory effects also probably contribute to the gene network. The cell-cycle regulator CDKN1C, encoded by an MEG, indirectly represses the phosphorylation and activity of the retinoblastoma-1 (RB1) protein, expressed from an MEG [61]. This finding is consistent with the co-adaptation theory's prediction of MEG–MEG interactions. CDKN1C expression itself is downregulated by IGF2 and PLAGL1/ZAC1 [57,62], both PEGs, which agrees with the predictions of the kinship theory.

Whether imprinted *trans* regulation occurs also through direct interactions between imprinted loci is unclear. Conventional ‘chromosome conformation capture' (3C) [63] identified specific chromatin loops at the *H19* locus and the advent of 4C widened the scope of known contacts [64]. The *H19* ICR was seen to interact with different chromosomes and, in a few cases, up to four contacts *in trans* were detected at once, although the vast majority turned out to be *cis* interactions. Both imprinted and non-imprinted gene interactions were detected, primarily in intergenic regions, which seemed to argue in favour of a complex transcriptional network [64]. However, Krueger & Osborne [65] underscore the idea that *trans* elements are a ‘common theme’ in mono-allelic gene expression because inter-chromosomal interactions are often linked to coordinated gene transcription through common usage of ‘transcription factories’ [66].

Regardless of their precise modes of action, the data suggest that imprinted *trans*-regulators do not influence the allele-specificity of imprinted gene expression or the establishment or maintenance of imprints. This contrasts with the situation in plants, particularly in *Arabidopsis*, where several imprinted genes encode chromatin repressors involved in the allelic repression of imprinted genes [1,2]. Rather, in mammals imprinted *trans*-regulators function to modulate levels of mRNA and protein produced from the already transcriptionally active allele.

## 6. Conclusion

Above we reviewed the imprinted gene network(s) and the rapidly growing literature on imprinted *trans* regulation in relation to evolutionary theories. About 10 imprinted *cis*- and *trans*-regulators of imprinted gene expression have been studied functionally thus far. Some seem to fulfil the predictions of the kinship theory (*IPW*, *Rtl1as/antiPeg11*). The effects of several others (e.g. PEG3, *Plagl1*/*Zac1* and *H19*), however, are harder to square with either the kinship or the co-adaptation theory, and other hypotheses may be required to complement the current theories. Systems biology approaches have provided novel insights and have pinpointed a large network of coordinately expressed imprinted and non-imprinted genes. This important discovery suggests many more regulatory links between imprinted genes than have been unravelled so far. Expression levels within the network as a whole are also strongly influenced by the state of the cell cycle and by the differentiation status of the cell. This may well be a confounding factor in explaining the effects due to alteration of individual imprinted *trans*-regulators. Overexpression of individual imprinted genes has indeed been shown to affect the cellular proliferation status of cultured cells, which, in turn, affects the levels of gene expression in the imprinted gene network [19,32]. Above, we discussed novel concepts that have emerged in imprinted gene regulation in mammals in the light of evolutionary theories that bear on the topic. The challenge will now be to obtain further insights into the interdependence of imprinted gene expression, into the biological processes to which these links are important, and into their evolutionary conservation.

The growing evidence for *trans*-regulatory interactions between imprinted genes raises the additional question as to whether these contribute to phenotypic abnormalities in embryos obtained by crossing closely related (sub)species. Some evidence for this has been obtained from crosses between different mouse species [67]. The co-adaptation theory, with its emphasis on epistatic interactions between genes, sees an obvious connection between imprinted genes and speciation, which typically requires a breakdown of epistatic interactions [68], while the kinship theory suggests that these imprinted *trans-*regulatory effects are evolving under conflict, producing just the type of perpetual evolutionary force capable of promoting reproductive isolation [69]. Further studies are required, here as well, to assess to what extent regulatory interactions within the imprinted gene network(s) in mammals constitute an evolutionary barrier against hybridization. Research on artificially induced hybrids in *Arabidopsis* suggests that imprinted genes could also contribute similarly to speciation in seed plants [70], although the available evidence remains limited.
